# Resistance of *Acinetobacter baumannii* Complex Clinical Isolates to Sulbactam–Durlobactam: A Systematic Review of Data from In Vitro Studies

**DOI:** 10.3390/pathogens14101062

**Published:** 2025-10-20

**Authors:** Matthew E. Falagas, Laura T. Romanos, Dimitrios Ragias, Charalampos Filippou

**Affiliations:** 1 Alfa Institute of Biomedical Sciences (AIBS), 9 Neapoleos Street, 151 23 Marousi, Greece; l.romanos@aibs.gr (L.T.R.); d.ragias@aibs.gr (D.R.); 2 School of Medicine, European University Cyprus, 6 Diogenous Str., 2404 Nicosia, Cyprus; c.filippou@euc.ac.cy; 3 Department of Medicine, Tufts University School of Medicine, 145 Harrison Ave, Boston, MA 02111, USA

**Keywords:** sulbactam–durlobactam, ETX2514, *A. baumannii* complex, *A. baumannii*, *A. calcoaceticus*, *A. nosocomialis*, *A. pittii*, pneumonia, hospital-acquired bacterial pneumonia, ventilator-associated bacterial pneumonia

## Abstract

Introduction: Due to the limited therapeutic options for patients with *Acinetobacter baumannii* complex infections, a new combination antimicrobial agent, sulbactam–durlobactam, has been developed. In this systematic review, we evaluated the available data on the resistance of *A. baumannii* complex clinical isolates to sulbactam–durlobactam. Methods: We performed a thorough search of four databases for relevant studies. The Clinical and Laboratory Standards Institute (CLSI) sulbactam–durlobactam breakpoint for *A. baumannii* complex susceptibility was used (MIC value ≤4 mg/L). Data on the presence of genes of various β-lactamases were also analyzed. Results: From 182 identified articles, 84 were thoroughly screened. Data extraction was performed on 20 articles (published 2017–2025) reporting on a total of 10,412 *A. baumannii* complex clinical isolates. Among the various β-lactamases genes present, the OXA subvariants OXA-23/OXA-23-like were the most common (in 561 isolates). The proportions of non-selected (consecutive) *A. baumannii* isolates found to be resistant to sulbactam–durlobactam were 1.2%, 1.2%, and 4.6% in the three studies, and with non-susceptibility (resistance and intermediate resistance) were 2%, 2.1%, and 4.6% in three other studies. Non-susceptibility was very rare among *A. calcoaceticus*, *A. nosocomialis*, and *A. pittii* isolates (0%, 0.3%, and 0.6%, respectively). The proportion of carbapenem-resistant *A. baumannii* isolates with resistance was 0–5.2%. The proportion of *A. baumannii* isolates selected for their reduced susceptibility profile (including reduced susceptibility to cefiderocol) with resistance was 1.4–27.3%. Discussion: The low proportion of sulbactam–durlobactam resistance among *A. baumannii* complex isolates supports the consideration of the use of this new antibiotic for its approved indications.

## 1. Introduction

*Acinetobacter baumannii* is a Gram-negative, lactose non-fermenting bacterium. Until approximately 20 years ago, it was considered primarily a colonizer rather than a significant cause of infection. Additionally, before that time, it was believed that infections caused by *A. baumannii* were not associated with increased mortality. It was often stated that patients ‘die with *A. baumannii* infection, not from it’, reflecting a belief that *A. baumannii* did not increase mortality. However, data from around the world eventually showed that *A. baumannii* infections can directly cause significant morbidity and mortality [1,2]. Nowadays, it is recognized that *A. baumannii* infections are frequently multidrug-resistant (MDR), extensively drug-resistant (XDR), or pandrug-resistant (PDR) and have disseminated globally [3,4].

As a result, therapeutic options for *A. baumannii* infections are now limited due to the extensive antimicrobial resistance of these pathogens. Older antibiotics, such as polymyxins (colistin and polymyxin B) and aminoglycosides, are frequently used despite their considerable nephrotoxicity. In addition, some broad-spectrum β-lactams with activity against *Pseudomonas aeruginosa* (piperacillin–tazobactam, ceftazidime, aztreonam, meropenem) and quinolones (e.g., levofloxacin) may exhibit activity against certain *A. baumannii* isolates. Although newer antibiotics that are modifications of tetracyclines (such as omadacycline and eravacycline) and tigecycline (a glycylcycline) exhibit promising activity against a variety of pathogens, their effectiveness against *A. baumannii* is relatively low and variable [5]. Newer β-lactam/β-lactamase inhibitor combinations (meropenem–vaborbactam, imipenem–relebactam, aztreonam–avibactam) have limited or variable activity against *A. baumannii* [6,7].

Another commonly used antibiotic combination for treating patients with *A. baumannii* infections is ampicillin–sulbactam [8]. Recent studies have shown an increase in bacterial resistance to this combination of agents, reinforcing the need for new agents and combinations to enter the market [9]. Increasing the sulbactam dose in this combination could potentially reduce the emergence of resistance to this combination in *A. baumannii*. However, there would not be a significant advantage to using a high-dose ampicillin–sulbactam combination for treatment, as there is considerable overlap between sulbactam resistance and carbapenem resistance [10]. Additionally, there is limited information on the clinical use of trimethoprim–sulfamethoxazole for the treatment of patients with *A. baumannii* infections [11]. *A. baumannii* isolates intrinsically produce several types of β-lactamases, especially Class C β-lactamases, according to the Ambler classification [such as *Acinetobacter*-derived cephalosporinases (ADC)] [12]. Additionally, *A. baumannii* isolates may harbor the oxacillinase-51 (OXA-51) gene. However, both ADC and OXA-51 genes are expressed at low levels, leading to low or no production of the relevant cephalosporinases (ADC) and carbapenemase (OXA-51) [12]. The expression of these genes may be promoted by the insertion sequence ISAba1, a genetic element that leads to subsequent gene overexpression [13]. *A. baumannii* can also horizontally acquire Ambler Class D (such as OXA-23, OXA-24, and OXA-58) carbapenemase genes, as well as Ambler Class B carbapenemases such as imipenemase (IMP), Verona integron-encoded metallo-β-lactamase (VIM), and New Delhi metallo-β-lactamase (NDM) [14,15]. *A. baumannii* isolates may also employ various mechanisms of resistance, such as loss of outer membrane permeability and the overexpression of efflux pumps, in addition to β-lactamase acquisition [14]. Clones with multiple mechanisms of resistance have been successful in dissemination. The IC2 clone (GC2), also known as ST2 based on the multilocus sequence typing (MLST) of the Pasteur scheme, and including ST208 and ST281 MLST types of the Oxford scheme, has disseminated globally [16,17]. It is a frequently isolated clone of *A. baumannii,* especially in patients with MDR healthcare-associated infections. The isolates of this clone may carry genes for the production of OXA-23, OXA-66, and carbapenemases [18].

To address the challenge of MDR *A. baumannii*, a new β-lactam/β-lactamase inhibitor combination, sulbactam–durlobactam (formerly ETX2514), was developed. This drug, with the market name Xacduro, was approved by the Food and Drug Administration (FDA) in 2023 for the treatment of adult patients with hospital-acquired bacterial pneumonia and ventilator-associated bacterial pneumonia (HABP/VABP) caused by isolates of the *A. baumannii* complex. The drug was fast-tracked and prioritized for review as a pathogen-specific antimicrobial agent [19]. From this perspective, sulbactam–durlobactam is a rare example of an antimicrobial approved for a specific pathogen. Another relevant example is fidaxomicin, which is currently approved only for the treatment of *Clostridioides difficile* disease.

Sulbactam–durlobactam is a targeted β-lactam/β-lactamase inhibitor (BL/BLI) combination. Sulbactam has a dual role: it acts as a β-lactam antibiotic (binding to penicillin-binding proteins 1 and 3) and also as a β-lactamase inhibitor [20]. Sulbactam is still susceptible to hydrolysis by certain β-lactamases, for example, TEM-1. In fact, one study showed that *A. baumannii* isolates carrying the TEM-1 β-lactamase gene had significantly higher MIC values in comparison to those that were negative for this gene. For this reason, it is paired with durlobactam, a novel diazabicyclooctane β-lactamase inhibitor [21]. Durlobactam is a novel diazabicyclooctane compound and has a broad spectrum of activity against several β-lactamases, namely, Class A, C, and D serine β-lactamases, according to the Ambler classification [22].

In the context of therapeutic developments for treating patients with *A. baumannii* infections, in this systematic review, we sought to evaluate the published evidence on the resistance of *A. baumannii* complex isolates to sulbactam–durlobactam. These data will help in decision-making regarding the appropriate use of the new combination antibiotic in clinical practice. Given the recent approval of sulbactam–durlobactam, understanding the current extent of any resistance to this agent is critical for its optimal use.

## 2. Methods

### 2.1. Sources and Eligibility Criteria

We conducted this systematic review in accordance with the Preferred Reporting Items for Systematic Reviews and Meta-Analyses (PRISMA) guidelines. The protocol of the study was not uploaded to a registry. A comprehensive literature review was conducted across four databases (Embase, PubMed, Scopus, and Web of Science) from their inception to 19 July 2025. Eligible for assessment were studies of any primary research design that met the following inclusion criteria: (a) the terms sulbactam–durlobactam included in the title/abstract/keywords, and (b) the terms minimum inhibitory concentration (MIC) or disk diffusion susceptibility testing data present.

The exclusion criteria were (a) non-primary research articles; (b) studies of isolates obtained from animal sources; (c) case reports focusing on a single patient or a single bacterial isolate; (d) primary research articles that did not contain relevant data for this review; (e) studies that did not contain data on susceptibility of *A. baumannii* complex isolates to sulbactam–durlobactam; (f) conference abstracts; and (g) studies evaluating ≤ 5 total isolates, for the sulbactam–durlobactam susceptibility testing.

### 2.2. Search Strategy and Screening of Studies

The detailed search strategy is presented in Supplementary File S1. Terms such as “sulbactam–durlobactam”, “resistance”, “non-susceptibility”, “MIC”, and “disk diffusion” were used. Additional articles were identified through manual screening of reference lists. Duplicate articles were removed using the Rayyan tool’s automatic DOI-based deduplication. We screened all retrieved studies in full text.

### 2.3. Breakpoints of Susceptibility Testing

At the time of writing, clinical breakpoints for sulbactam–durlobactam against *A. baumannii* complex have exclusively been published by the Clinical and Laboratory Standards Institute (CLSI). Isolates are considered susceptible (S) if the sulbactam minimal inhibitory concentration (MIC) is ≤4 mg/L with a fixed 4 mg/L durlobactam concentration. Isolates with a sulbactam MIC of ≥16 mg/L are categorized as resistant (R). Isolates with a sulbactam MIC of 8 mg/L are classified as intermediate resistant (I). The term ‘non-susceptible’ refers to any isolate with MIC > 4 mg/L (thus categorized as intermediate or resistant under CLSI criteria).

### 2.4. Data Extraction

Our analysis includes data on the total number of the studied isolates, the number of isolates of each species, and the presence of various β-lactamases (based on phenotypic and/or genotypic methods). Also included are data on the MIC range (mg/L), MIC_50_, MIC_90_, and the percentage of resistance among the studied isolates. Two investigators (L.T.R. and D.R.) independently performed the study selection, screening, and data extraction. Any discrepancies were resolved with the assistance of a senior investigator (M.E.F.).

### 2.5. Data Tabulation

The data were categorized according to bacterial species and the presence of β-lactamase genes. For each group, the following information was recorded: the number of specific isolates, the presence of β-lactamase genes, MIC ranges in milligrams per liter (mg/L), and the percentage of isolates resistant to sulbactam–durlobactam. Resistance was determined based on the criteria set by the respective studies using susceptibility breakpoints defined by the CLSI.

## 3. Results

### Selection of Relevant Articles

**Figure 1:**
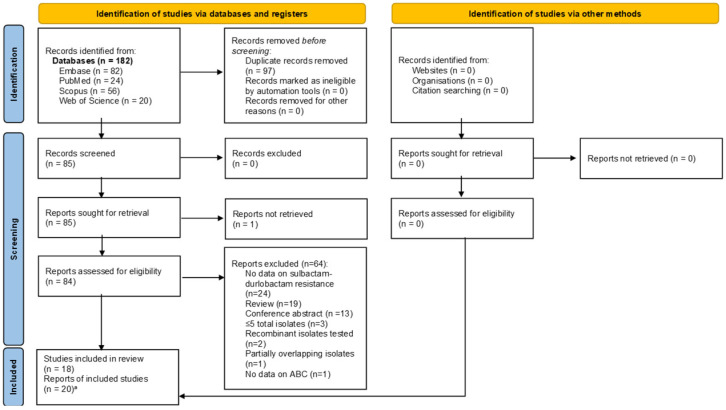
“Preferred Reporting Items for Systematic Reviews and Meta-Analyses” (PRISMA) flow diagram for identifying, screening and selecting articles. (Source: [42]). Note: ^a^ There were two separate reports of two different studies.

The PRISMA reporting checklist for the abstract and the text is shown in Supplementary Files S2 and S3. In Figure 1, the PRISMA flow diagram is shown. It describes the evaluation, selection, and inclusion of the relevant articles. A total of 182 articles were identified. After removing duplicates, 85 unique articles remained. We obtained 84 of these in full-text and assessed them (one potentially relevant article could not be retrieved in full text), ultimately including 20 articles in our analysis. These 20 articles corresponded to 18 unique studies [10,23,24,25,26,27,28,29,30,31,32,33,34,35,36,37,38,39,40,41]. In two cases, two articles reported separate data from a single study [29,31] and [28,32].

The included in vitro studies, published between 2017 and 2025, reported results on a total of 10,412 isolates (Table 1). The articles collectively reported on various *A. baumannii* complex isolates, including *A. baumannii* (10 studies) [10,23,25,26,27,28,30,32,34,39,40], *A. calcoaceticus* (2 studies) [28,30,32], *A. nosocomialis* (2 studies) [28,30,32], and *A. pittii* (2 studies) [28,30,32]. Seven articles specifically included data on carbapenem-resistant *A. baumannii* (CRAB) isolates [23,24,33,35,37,38,41].

The studied isolates had the presence of various β-lactamases genes, particularly oxacillinase (OXA), Temoniera β-lactamase (TEM), and *Acinetobacter*-derived cephalosporinase (ADC). Among the OXA subvariants, OXA-23 (including OXA-23-like variants) was the most common OXA gene (present in 561 isolates), followed by OXA-66-like (136 isolates), OXA-69-like (80), and OXA-51-like (70 isolates). TEM-1 was the predominant TEM variant (179 isolates). The most frequent ADC genes were ADC-30 and ADC-73 (present in 60 and 67 isolates, respectively).

In all studies, the CLSI breakpoints for susceptibility were used to determine the percentage of isolates resistant to sulbactam–durlobactam [10,23,24,25,26,27,28,29,30,31,32,33,34,35,36,37,38,39,40,41]. In Table 1, we present the in vitro susceptibility testing results of non-selected (consecutive) *A. baumannii* complex isolates. The percentage of non-selected *A. baumannii* isolates that were resistant was 1.2%, 1.2%, and 4.6% in three studies [10,25,27]. The percentages with intermediate resistance in these studies were 1.9%, 7.1%, and 2%, respectively [10,25,27]. The percentage of non-selected (consecutive) *A. baumannii* isolates with non-susceptibility (resistance and intermediate-resistance) in three other studies was 2%, 2.1%, and 4.6%; the relevant data were presented in five articles [28,29,31,32,40].

Non-susceptibility was very rare in *A. calcoaceticus, A. nosocomialis,* and *A. pittii* (0%, 0.3%, and 0.6% of isolates, respectively) in the study that reported data for these species [28,32] (Table 1). In one study that did not specify the *Acinetobacter* species, the resistance of isolates to this drug was reported at 3%, and intermediate resistance was reported at 0% [36] (Table 1). Another study displayed the non-susceptibility percentage for all *A. baumannii* complex isolates studied combined as 2.3% [30].

In studies focusing on selected CRAB isolates (Table 2), resistance ranged from 0% to 5.2%, and intermediate resistance ranged from 0% to 12% [23,24,33,35,37,38,41].

In Table 3, we present the in vitro susceptibility testing results of selected (non-consecutive) *A. baumannii* complex isolates. In a 2023 study, eleven *A. baumannii* clinical isolates showing reduced susceptibility to cefiderocol (MIC value ≥ 1 mg/L) were analyzed. Notably, 27.3% (3 of 11) of those isolates were resistant to sulbactam–durlobactam [39]. Another 2023 study examined the resistance of 10 isolates, which had been previously characterized by whole-genome sequencing. It found that 10% of isolates were resistant and 20% were intermediate to sulbactam–durlobactam [34].

A 2022 study examined 100 non-duplicate clinical *A. baumannii* isolates, which had previously been characterized for resistance mechanisms representative of MDR patterns commonly observed in *A. baumannii*. They exhibited high non-susceptibility to other antibiotics (e.g., 82% to amikacin and 95% to cefepime). Against sulbactam–durlobactam, 15% of these isolates were fully resistant, and 4% were intermediate. Notably, in the subset of five NDM-producing isolates, 80% were resistant to sulbactam–durlobactam [26].

A 2019 study evaluated 72 genotypically characterized *A. baumannii* isolates (all carrying multiple Class C and D β-lactamase genes). It found resistance and intermediate proportions of 1.4% and 4.2%, respectively [23].

## 4. Discussion

Our analysis indicates that sulbactam–durlobactam has high activity against *A. baumannii* complex clinical isolates overall. The observed resistance proportions were low despite the notoriously difficult-to-treat nature of *A. baumannii*—with the notable exception of NDM-producing isolates.

The FDA approved sulbactam–durlobactam for the treatment of adult patients with HABP/VABP caused by isolates of the *A. baumannii* complex in a fixed 1:1 ratio (1 g of each agent per dose), to be administered via intravenous infusion over 3 h. The recommended treatment duration is 7 to 14 days, depending on the patient’s clinical status. For the first three doses, the drug should be administered every 12 h; thereafter, it should be administered every 24 h. Dose adjustments should be made for patients with reduced renal function.

Various Phase 1 clinical trials (NCT02971423, NCT03303924, NCT03310463) were conducted to assess the safety, tolerability, and pharmacokinetic characteristics of sulbactam–durlobactam on healthy subjects [43,44,45]. Another Phase 1 clinical trial (NCT06801223) is currently recruiting to evaluate the activity of this antimicrobial combination in pediatric patients with infections caused by the *A. baumannii* complex [46].

One Phase 2 clinical trial (NCT03445195) was also completed to assess the safety, tolerability, and pharmacokinetic characteristics of this drug on hospitalized adult patients with complicated urinary tract infections [47]. One study arm received sulbactam–durlobactam 1:1 (1 g of each agent) infused over 3 h, every 6 h, while the other study arm received 1 g of a placebo intravenously. Both arms also received 500 mg of IV imipenem–cilastatin every 6 h. In an analysis of data from 68 patients of this trial, no significant difference was observed between the compared arms (76.6% and 81% for the sulbactam–durlobactam and the placebo group, respectively, for the overall success, defined as clinical cure and microbiologic eradication in the microbiologically modified intent-to-treat population) [48].

A Phase 3 clinical trial (NCT03894046) was also conducted to evaluate the efficacy and safety of the intravenous administration of the sulbactam–durlobactam combination for treating patients with *A. baumannii* complex infections. This trial was named ATTACK. The comparator agent was colistin. Overall, it met the non-inferiority criteria versus the comparator in terms of 28-day all-cause mortality (19.0% vs. 32.3%) and showed lower nephrotoxicity in the first 28 days after treatment (13.2% vs. 37.6%) [49].

Additionally, an observational study (NCT06746883) is currently recruiting. The aim is to assess the safety and risk of hypersensitivity reactions (including anaphylaxis) to sulbactam–durlobactam in adults with infections caused by the *A. baumannii* complex. It will monitor the occurrence of adverse events over 28 days [50].

Given the in vitro antimicrobial susceptibility data evaluated in this article, further studies should explore the antimicrobial activity of sulbactam–durlobactam against other Gram-negative bacteria. A 2021 study evaluated the activity of sulbactam–durlobactam against various isolates of the *Burkholderia* species. Among 150 *Burkholderia cepacia* complex and *Burkholderia gladioli* isolates, 12.7% of non-susceptibility was observed when the MIC ≤ 4 mg/L susceptibility breakpoint was utilized [51].

Our study has strengths and limitations. We conducted a thorough literature search across four databases and employed a transparent study selection process in our analysis, adhering to the PRISMA guidelines. This led to a comprehensive evaluation of the published literature on the resistance of *A. baumannii* isolates, building upon a prior systematic review [22].

However, we did not register the research protocol for our study in a relevant depository and did not perform a risk of bias assessment of the included studies as there is a lack of a globally accepted and validated tool for risk of bias assessment of in vitro antimicrobial susceptibility studies. Additionally, we did not report on other resistance mechanisms besides the presence of β-lactamase genes (that may not be fully expressed and, thus, may not lead to the production of β-lactamases). The role of these mechanisms in antimicrobial resistance is not negligible. Various studies included in our analysis mentioned the coexistence of other resistance mechanisms, such as insertion mutations in certain protein targets and changes in penicillin-binding proteins (PBPs). This aligns with recent molecular studies identifying Class B β-lactamases (NDM and other metallo-β-lactamases) and PBP3 amino acid insertions/mutations as key drivers of sulbactam–durlobactam resistance [32].

Future studies should explore the antimicrobial activity of sulbactam–durlobactam on other Gram-negative bacteria, including Enterobacterales, and lactose non-fermenting Gram-negative bacteria beyond *A. baumannii* complex, specifically *Burkholderia cepacia* complex [51]. Additionally, the activity of sulbactam–durlobactam against MBL-producing Gram-negative bacteria should be further studied, given the increasing prevalence of MBL-producing *A. baumannii* (e.g., NDM) and the poor activity of sulbactam–durlobactam against such strains [26]. Notably, recent clinical guidelines have incorporated sulbactam–durlobactam as a preferred treatment for carbapenem-resistant *A. baumannii* infections, underscoring the importance of ongoing surveillance for resistance as this agent enters wider use [52].

## 5. Conclusions

The evaluation of published in vitro antimicrobial susceptibility studies of *A. baumannii* complex clinical isolates to sulbactam–durlobactam demonstrates low resistance. These data, together with the efficacy results of the relevant clinical trials, suggest that the new combination antimicrobial drug should be considered for treating patients with *A. baumannii* complex infections. The occasional occurrence of resistance to sulbactam–durlobactam among the studied isolates and the considerable proportion of resistance among selected *A. baumannii* complex clinical isolates with advanced resistance profiles necessitate the review of the results of appropriate in vitro antimicrobial susceptibility testing for the use of the new antibiotic in clinical practice.

## Figures and Tables

**Table 1:** Proportion of resistance of non-selected (consecutive) *Acinetobacter baumanni* complex to sulbactam/durlobactam.

Author ^a^	Year	Isolates	N	β-Lactamase Genes (Number of Isolates)	MIC Value or Range (mg/L)	MIC_50_ (mg/L)	MIC_90_ (mg/L)	Resistance % (I: %) ^b^ [Breakpoint] ^c^
Buyukyanbolu [10]	2025	*A. baumannii*	523	OXA-23 (8), OXA-66 (8), ADC-73 (7), TEM-1 (3), ADC-56 (2), OXA-72 (2), ADC-222 (2), OXA-24 (2), OXA-95 (2), NDM-1 (2), NDM-5 (2), OXA-23 [M1X] (1), OXA-66 [T10K] (1), ADC-76 (1), OXA-58 (1), OXA-68 (1)	≤0.125–>64	2	4	1.2 (I: 1.9)
Iovleva [27]	2024	*A. baumannii*	87	OXA-82 (65), OXA-23 (59), OXA-66 (8), OXA-95 (6), OXA-24 (5), OXA-83 (3), OXA-51 (2), OXA-223 (2), OXA-72 (1), OXA-113 (1), OXA-166 (1)	0.5–64	2	8	4.6 (I: 2)
Miller [31] Kaye [29]	2024 2023	*A. baumannii* complex	175	OXA-23 (8), OXA-66 (6), TEM-1 (5), ADC-30 (3), ADC-73 (3), ADC-115 (1), ADC-6-like (1), OXA-80 (1), OXA-71 (1)	0.25–32	2	4	4.6 ^d^
Senterre-Henriksen [36]	2024	*Acinetobacter* spp.	501	OXA (202), MBL (3), OXA + MBL (9), OXA + ESBL (4), ESBL + KPC (1), No Acquired β-lactamase (8)	NA	NA	4	3 (I: 0)
Moussa [32] Karlowsky [28]	2023 2022	All *A. baumannii* *A. calcoaceticus* *A. nosocomialis* *A. pittii*	5032 4038 55 296 638	OXA-23 (34), NDM-1 (32), OXA-66 (32), ADC-30 (11), ADC-73 (11), OXA-24 (9), OXA-58 (6), ADC-25 (5), OXA-69 (5), ADC-169 (4), OXA-402 (4), CTX-M-15 (3), ADC-26 (3), CARB-2 (3), OXA-94 (3), TEM-1 (19), VEB-1 (2), ADC-176 (2), ADC-30-like (2), ADC-99-like (2), ADC-43-like (2), OXA-533-like (2), OXA-64 (2), OXA-71 (2), ADC-11 (1), ADC-131-like (1), ADC-152 (1), ADC-163 (1), ADC-169-like (1), ADC-176-like (1), ADC-18 (1), ADC-181 (1), ADC-214 (1), ADC-216 (1), ADC-39 (1), ADC-43 (1), ADC-5 (1), ADC-53 (1), ADC-6-like (1), ADC-7-like (1), ADC-73-like (1), ADC-76 (1), ADC-80 (1), ADC-82 (1), ADC-91 (1), ADC-97-like (1), CARB-16 (1), OXA-10 (1), OXA-121 (1), OXA-132 (1), OXA-23+ (1), OXA-259 (1), OXA-407 (1), OXA-500 (1), OXA-51 (1), OXA-65 (1), OXA-66+ (1), OXA-68 (1), OXA-70 (1), OXA-72 (1), OXA-820 (1), OXA-83 (1), OXA-91 (1), PER-1 (1), PER-7 (1), VIM-4 (1)	≤0.03–>64 ≤0.03–>64 0.12–2 ≤0.03–8 ≤0.03–32	1 1 0.5 0.5 0.5	2 2 1 1 2	1.7 ^d^ 2 ^d^ 0 0.3 ^d^ 0.6 ^d^
McLeod [30]	2020	*A. baumannii* complex *A. baumannii* *A. calcoaeceticus* *A. nosocomialis* *A. pittii*	1722 1420 10 60 232	OXA-23 (17), OXA-66 (15), NDM-1 (11), TEM-1 (8), ADC-25 (5), ADC-73 (5), ADC-30 (4), OXA-24 (4), OXA-58 (3), ADC-152 (S341T) (2), ADC-169 (2), CARB-2 (2), OXA-64 (2), OXA-69 (2) OXA-132 (2), OXA-402 (2), ADC-5 (1), ADC-5 (G239S, N341T) (1), ADC-7-like (1), ADC-26 (1), ADC-50 (1), ADC-53 (A236V) (1), ADC-80 (V119E) (1), ADC-82 (1), ADC-97-like (1), ADC-99-like (1), ADC-176 (1), CTX-M-15 (1), OXA-64 (1) OXA-65 (1), OXA-70 (1), OXA-71 (1), OXA-83 (1), OXA-91(1), OXA-94 (2), PER-7(1), subclass B3 MBL (1), VEB-1 (1)	≤0.03–>64 ≤0.03–>64 0.12–1 0.12–4 0.12–4	1 1 0.5 0.5 0.5	2 4 1 1 2	2.3 ^d^ NA
Yang [40]	2020	*A. baumannii*	982	NA	≤0.03–>64	1	2	2.1 ^d^
Durand-Réville [25]	2017	*A. baumannii*	84	OXA-23 (49), OXA-66 (48), TEM-1 (41), ADC-30 (18), ADC-73 (18), OXA-40 (7), ADC-82 (6), OXA-72 (6), OXA-65 (5), OXA-113 (5), ADC-ETX1 ^e^ (4), ADC-76 (4), OXA-68 (4), ADC-11 (3), OXA-10/69 (3), OXA-58 (3),SHV-5 (3), ADC-ETX29 (2), ADC-25 ^e^ (2), ADC-79 (2), ADC-ETX15 (2), OXA-20 (2), OXA-51 (2), OXA-64 (2), OXA-65 (2),OXA-71 (2), OXA-82 (2), OXA-132 (2), PER-1 (2), GES-12 (1), IMP-4(b) (1), ADC-1 (1), ADC-26 (1), ADC-80 (1), ADC-87 ^e^ (1), ADC-ETX3 ^f^ (1), ADC-ETX5 ^f^ (1), ADC-ETX7 (1), ADC-ETX8 (1), ADC-ETX9 (1), ADC-ETX10 ^e^ (1), ADC-ETX12 ^e^ (1), ADC-ETX13 (1), ADC-ETX17 (1), ADC-ETX18 (1), ADC-ETX19 ^e^ (1), ADC-ETX20 (1), ADC-ETX21 (1), ADC-ETX22 (1), ADC-ETX26 (1), ADC-ETX27 (1), ADC-ETX33 (1), OXA-40 (1), similar to ADC-52 (1), OXA-69 (1), OXA-73 (1), OXA-94 (1), OXA-100 (1), OXA-109 (1), OXA-172 (1), OXA-398 (1), OXA-ARC2597 (1), OXA-ARC2598 (1), OXA-ARC2719 (1), OXA-ARC3488 (1), OXA-ARC3489 (1), PER-unq (1), SHV-12 (1)	0.25–16	2	4	1.2 (I: 7.1)

Notes: ^a^ Studies are presented in descending chronological order (and alphabetical order within a year); ^b^ I = Intermediate resistance; ^c^ According to the criteria, as defined by the authors in each study; ^d^ non-susceptibility; ^e^ original spectrum; ^f^ spectrum undefined. Abbreviations: *A. baumannii*, *Acinetobacter baumannii*; ABC, *A. baumannii*–*calcoaceticus* complex, *A. calcoaeceticus*, *Acinetobacter calcoaeceticus*; ADC, *Acinetobacter*-derived cephalosporinase (Class C β-lactamase); *A. nosocomialis*, *Acinetobacter nosocomialis*; *A. pitti*, *Acinetobacter pitti*; CARB, carbenicillinase (Class A β-lactamase); CTX-M, cefotaximase-Munich (Class A extended-spectrum β-lactamase); ESBL, extended-spectrum β-lactamase (hydrolyzes extended-spectrum cephalosporins and monobactams); GES, Guiana extended spectrum β-lactamase (Class A β-lactamase, includes ESBLs and some carbapenemases); IMP, imipenemase (Class B metallo-β-lactamase); KPC, Klebsiella pneumoniae carbapenemase (Class A carbapenemase); MBL, metallo-β-lactamase (Class B β-lactamase, hydrolyzes carbapenems); MIC, minimum inhibitory concentration; NA, Not applicable/Not available; NDM, New Delhi metallo-β-lactamase (Class B carbapenemase); OXA, oxacillinase (Class D β-lactamase, many variants act as carbapenemases); PER, Pseudomonas extended resistance (Class A extended-spectrum β-lactamase); SHV, sulfhydryl variable β-lactamase (Class A β-lactamase, includes penicillinases and ESBLs); TEM, Temoniera β-lactamase (Class A β-lactamase, includes penicillinases and ESBLs); VEB, Vietnam extended spectrum β-lactamase (Class A extended-spectrum β-lactamase); VIM, Verona integron-encoded metallo-β-lactamase.

**Table 2:** Proportion of resistance of carbapenem-resistant *Acinetobacter baumannii* to sulbactam/durlobactam.

Author ^a^	Year	Isolates	N	β-Lactamase Genes (Number of Isolates)	MIC Value or Range (mg/L)	MIC_50_ (mg/L)	MIC_90_ (mg/L)	Resistance % (I: %) ^b^ [Breakpoint] ^c^
Doragio [24]	2025	CRAB	58	OXA-51-like (58), OXA-23-like (49), ADC-30 (20), ADC-73 (20), TEM-1D (18), OXA-24-like (9), ADC-33 (5), ADC-56 (4), ADC-222 (4), ADC-268 (2), ADC-103 (1), ADC-229 (1), OXA-50-like (1), PAO (1)	0.5–32	2	8	5.2 (I: 12)
Zalacain [41]	2024	CRAB	340	NA	0.25–>32	2	4	3.8 (I: 3.2)
Petropoulou [35]	2022	CRAB	190	TEM-1 + NDM-1 + ADC-73 + OXA-23 + OXA-66 (1), TEM-1 + ADC-73 + OXA-23 + OXA-66 (1), ADC-188 + OXA-23 + OXA-66 (1)	0.5–>64	4	8	1.6 (I: 10.5)
Segatore [37]	2022	CRAB	141	ADC-25 + OXA-20 + OXA-58 + OXA-66 (7), ADC-25 + OXA-20 + OXA-58 (4)	0.06–>128	0.5	4	5 (I: 2.8)
Nodari [33]	2021	CRAB	112	OXA-24/40-like (48), OXA-23 (34), OXA-23 + OXA-24/40-like (17), OXA-143-like (10), OXA-23 + OXA-143-like (2), OXA-58 (1) (75 of the above isolates were also TEM-1 positive)	≤0.25–4	1	1	0 (I: 0)
Seifert [38]	2020	CRAB	246	OXA-23-like (184), OXA-40-like (47), OXA-58-like (3), IMP-26 (1), NDM-1 (3), OXA-51 (7), OXA-237 (1)	0.25–128	1	2	2.4 (I: 1.2)
Barnes [23]	2019	CRAB	26	NA	0.25–4	2	2	0 (I: 0)

Notes: ^a^ Studies are presented in descending chronological order (and alphabetical order within a year); ^b^ I = Intermediate resistance; ^c^ According to the criteria, as defined by the authors in each study. Abbreviations: ADC, *Acinetobacter*-derived cephalosporinase (Class C β-lactamase); CRAB, Carbapenem resistant *A. baumannii*; IMP, imipenemase (Class B metallo-β-lactamase); NA, Not applicable/Not available; NDM, New Delhi metallo-β-lactamase (Class B carbapenemase); OXA, oxacillinase (Class D β-lactamase, many variants act as carbapenemases); PAO, *Pseudomonas aeruginosa* strain (referring to enzymes characteristic of *P. aeruginosa*; TEM, Temoniera β-lactamase (Class A β-lactamase, includes penicillinases and ESBLs).

**Table 3:** Proportion of resistance of selected (non-consecutive) *Acinetobacter baumanni* complex to sulbactam/durlobactam.

Author ^a^	Year	Isolates	N	β-Lactamase Genes (Number of Isolates)	MIC Value or Range (mg/L)	MIC_50_ (mg/L)	MIC_90_ (mg/L)	Resistance % (I: %) ^b^ [Breakpoint] ^c^
Le Terrier [39]	2023	*A. baumannii*	11	OXA-23 (9), PER-7 (5), PER-1 (3), NDM-1 (2), NDM-5 (1)	0.25–128	1	128	27.3 (I: 0)
O’Donnell [34]	2023	*A. baumannii*	10	ADC-5 (1), ADC-11 (1), ADC-30 (2), ADC-33 (1), ADC-80 (1), ADC-82 (2), ADC-99 [N379S] (1), ADC-176 (1), ADC-214 [T341S] (1), OXA-23 (5), OXA-64 (1), OXA-65 (1), OXA-66 (2), OXA-66 [K42] (1), OXA-69 (1), OXA-72 (3), OXA-82 (1), OXA-83 (1), OXA-94 (1), OXA-259 (1), TEM-1 (4)	0.5–16	1	8	10 (I: 20)
Findlay [26]	2022	*A. baumannii*	100	OXA-23 (73), OXA-72 (10), OXA-40 (6), OXA-58 (5), OXA-24 (1), NDM-1 (4), NDM-5 (1)	0.06–64	4	16	15 (I: 4)
Barnes [23]	2019	*A. baumannii*	72	ADC (71), OXA-69-like (70), TEM (29), OXA-58-like (9), OXA-23-like (8), PER (2) In the 4 non-susceptible strains: ADC-25 + OXA-66 + TEM-1 (3), ADC-79 + OXA-66 + OXA-69 + TEM-1 (1)	0.5–32	1	2	1.4 (I: 4.2)

Notes: ^a^ Studies are presented in descending chronological order (and alphabetical order within a year); ^b^ I = Intermediate resistance; ^c^ According to the criteria, as defined by the authors in each study. Abbreviations: *A. baumannii*, *Acinetobacter baumannii*; ADC, *Acinetobacter*-derived cephalosporinase (Class C β-lactamase); MIC, minimum inhibitory concentration; NA, Not applicable/Not available; NDM, New Delhi metallo-β-lactamase (Class B carbapenemase); OXA, oxacillinase (Class D β-lactamase, many variants act as carbapenemases); PER, *Pseudomonas* extended resistance (Class A extended-spectrum β-lactamase); TEM, Temoniera β-lactamase (Class A β-lactamase, includes penicillinases and ESBLs).

## Data Availability

No new data were created or analyzed in this study.

## Supplementary Materials

The following supporting information can be downloaded at: https://www.mdpi.com/article/10.3390/pathogens14101062/s1. Supplementary File S1. Detailed search strategies used in each resource (as of 21 July 2025). Supplementary File S2. PRISMA 2020 for the abstract reporting checklist [42]. Supplementary File S3. PRISMA 2020 for the main article text reporting checklist [42].

## Abbreviations

*A. baumannii*	*Acinetobacter baumannii*
*A. calcoaceticus*	*Acinetobacter calcoaceticus*
*A. nosocomialis*	*Acinetobacter nosocomialis*
*A. pittii*	*Acinetobacter pittii*
ADC	*Acinetobacter*-derived cephalosporinases
β-lactamases	beta-lactamases
BL	β-lactam
BLI	β-lactamase inhibitor
CLSI	Clinical and Laboratory Standards Institute
CRAB	carbapenem-resistant *A. baumannii*
DOIs	digital object identifiers
FDA	Food and Drug Administration
HABP	hospital-acquired bacterial pneumonia
IMP	imipenemase
IV	intravenous
MBL	metallo-β-lactamase
MDR	multidrug-resistant
MIC	minimal inhibitory concentration
NDM	New Delhi metallo-β-lactamase
OXA	oxacillinase
PBP1 and PBP3	penicillin-binding proteins 1 and 3
PDR	pandrug-resistant
PRISMA	Preferred Reporting Items for Systematic Reviews and Meta-Analyses
TEM	Temoniera β-lactamase
VABP	ventilator-associated bacterial pneumonia
VIM	Verona integron-encoded metallo-β-lactamase
XDR	extensively drug-resistant
