# Supplementary material for: Resistance of Acinetobacter baumannii Complex Clinical Isolates to Sulbactam–Durlobactam: A Systematic Review of Data from In Vitro Studies
Source: Pathogens. 2025 Oct 20;14(10):1062. doi: 10.3390/pathogens14101062 (PMC12566789; doi:10.3390/pathogens14101062)
Supplement: Supplementary file 1 [file pathogens-14-01062-s001.zip › Supplementary file 1. Detailed search strategies.pdf]

Supplementary file 1. Detailed search strategies used in each resource as of 21 July 2025.

Resource	Search string	Coverage (years)	Results
Embase	("sulbactam-durlobactam" OR "sulbactam-ETX2514" OR (sulbactam AND durlobactam) OR (sulbactam AND ETX2514)) AND ("antibiotic resistance" OR resistance OR "non-susceptibility" OR "non susceptibility" OR nonsusceptibility OR "reduced susceptibility") AND (MIC OR "minimum inhibitory concentration" OR "disc diffusion")	1947 –present	82
PubMed	("sulbactam-durlobactam" OR "sulbactam-ETX2514" OR (sulbactam AND durlobactam) OR (sulbactam AND ETX2514)) AND ("antibiotic resistance" OR resistance OR "non-susceptibility" OR "non susceptibility" OR nonsusceptibility OR "reduced susceptibility") AND (MIC OR "minimum inhibitory concentration" OR "disc diffusion")	1946 –present	24
Scopus	("sulbactam-durlobactam" OR "sulbactam-ETX2514" OR (sulbactam AND durlobactam) OR (sulbactam AND ETX2514)) AND ("antibiotic resistance" OR resistance OR "non-susceptibility" OR "non susceptibility" OR nonsusceptibility OR "reduced susceptibility") AND (MIC OR "minimum inhibitory concentration" OR "disc diffusion")	1966 –present	56
Web of Science	("sulbactam-durlobactam" OR "sulbactam-ETX2514" OR (sulbactam AND durlobactam) OR (sulbactam AND ETX2514)) AND ("antibiotic resistance" OR resistance OR "non-susceptibility" OR "non susceptibility" OR nonsusceptibility OR "reduced susceptibility") AND (MIC OR "minimum inhibitory concentration" OR "disc diffusion")	1900 – present	20
